# Molecular typing and antimicrobial resistance profiling of 33 mastitis-related *Staphylococcus aureus* isolates from cows in the Comarca Lagunera region of Mexico

**DOI:** 10.1038/s41598-021-86453-2

**Published:** 2021-03-25

**Authors:** Y. Mora-Hernández, E. Vera Murguía, J. Stinenbosch, P. Hernández Jauregui, Jan Maarten van Dijl, G. Buist

**Affiliations:** 1 Department of Medical Microbiology, University of Groningen, University Medical Center Groningen, Hanzeplein 1, P.O. Box 30001, 9700 RB Groningen, The Netherlands; 2 Cyta Labs, Puebla, Mexico

**Keywords:** Bacteriology, Pathogens, Sequencing, Microbiology techniques, Infectious-disease epidemiology

## Abstract

Mastitis in cows is a major cause of economic losses and it is commonly associated with *Staphylococcus aureus*. Little is known about the *S. aureus* lineages causing mastitis in Mexican cattle. The aim of this study was to type *S. aureus* isolates causing mastitis in cows from the Comarca Lagunera region in Mexico in 2015–2016. Multi-locus variable number tandem repeat fingerprinting (MLVF) of 33 *S. aureus* isolates obtained from 210 milk samples revealed the MLVF clusters A (n = 1), B (n = 26), C (n = 5) and D (n = 1). *Spa*-typing showed that clusters A and B represent the *spa*-type t224, cluster C includes *spa*-types t3196 and t416, and cluster D represents *spa*-type t114. The different *spa*-types were mirrored by the masses of protein A bands as detected by Western blotting. Antimicrobial susceptibility testing showed that one isolate was susceptible to all antimicrobials tested, whereas all other strains were resistant only to benzylpenicillin. These findings show that only four *S. aureus* lineages, susceptible to most antimicrobials, were responsible for causing mastitis at the time of sampling. Lastly, many isolates carried the same small plasmid, designated pSAM1. The high prevalence of pSAM1 amongst the antimicrobial-susceptible isolates suggests an association with bovine colonization or mastitis rather than antimicrobial resistance.

## Introduction

Mastitis is the disease that causes the highest economic loss in the dairy industry worldwide^1^. The prevalence of mastitis amongst cattle in Mexico was reported to be up to 35%, depending on the region, the raining season and the system of cattle farming^2,3^.

Mastitis is an inflammation of the mammary gland, due to the invasion and destruction of milk-producing tissue by bacteria. This can be caused by Gram-positive and Gram-negative bacteria. The main reason for infection is poor hygiene due to which these bacteria are most often transmitted via milk machines, udder cloths, milkers’ hands, or calves^4^. The most common bacteria causing mastitis are staphylococci, streptococci, *Escherichia coli* and *Klebsiella pneumoniae*^5^. The Gram-positive bacterium *Staphylococcus aureus* is most frequently isolated from infected udders in dairy cattle^5^. Mastitis is usually treated with antimicrobials, such as penicillin G, cloxacillin, macrolides, lincosamides, and cephalosporins^6^.

Staphylococci are part of the normal bacterial flora of the skin, mucous membranes and urogenital tract in mammals and birds^7^. *S. aureus* is a facultative anaerobic bacterial pathogen carried by humans and many different mammals. It is currently not well understood what makes these bacteria switch from a commensal to a pathogenic lifestyle in susceptible individuals. However, once *S. aureus* has breached the skin or mucosal barriers, it can infect almost every part of the human body. *S. aureus* can also cause various types of infections in domestic animals^8^. In veterinary medicine, *S. aureus* is notorious for causing mastitis in cattle, sheep, goats, and horses; dermatitis in sheep and goats; botryomycosis in pigs and horses; and comb necrosis, bacterial chondronecrosis, and septicemia in poultry^7^. Boss et al. demonstrated that in animals the majority of *S. aureus* strains evolved from human strains. For example, strains with the sequence type 8 (ST8) and strains belonging to the clonal complexes (CC) CC5, CC8, CC59, CC97, and CC398 encountered in cows were of human origin^9^.

Resistant *S. aureus* can easily be transmitted between humans and animals, as has been shown for strains with the ST398 that were transmitted from pigs to humans resulting in major health problems^7^. However, the zoonotic transfer of *S. aureus* from milk and intramammary infections to humans seems to be very low^9^.

To determine the level of bacterial spread among animals and humans, various typing methods are used. As such, the distinction of organisms within a species by typing has become a very important epidemiological tool. The currently available typing methods can be classified into phenotyping and genotyping (“molecular” typing), the latter one being the more sensitive and more appropriate approach to study the bacterial population genetics^10^.

Several typing methods have been described to genotype *S. aureus*^11^. Pulsed-field gel electrophoresis (PFGE) was for many years considered as the “gold standard” among the typing methods, because it has a high power of discrimination, is highly reproducible and not expensive. However, this technique is labor-intensive, subjective and technically demanding^11^. Today, whole-genome sequencing (WGS) is gradually becoming the new gold standard for typing and studying bacterial population genetics, especially because it has a much higher discriminatory power than any other bacterial typing technique. Nevertheless, there is still a need to standardize and unify data obtained from typing tools based on WGS for straightforward inter-laboratory comparisons^12^. The latter issue has been overcome in another commonly applied sequencing-based *S. aureus* typing technique named *spa*-typing, which involves the sequencing of the repeat region of the *spa* gene encoding the immunoglobulin G-binding protein A^13^. Although this method has less discriminatory power than PFGE and WGS, there are several advantages that make it one of the most frequently used typing techniques for the classification of *S. aureus.* These advantages include high reproducibility, low costs, user friendliness, quickness, portability of data and high-throughput^11^. Another convenient method to type *S. aureus* isolates is multi-locus variable number tandem repeat (VNTR) fingerprinting (MLVF), which consists of a multiplex PCR-based assay to determine the polymorphism of VNTRs in 7 individual genes of *S. aureus* (*sspA*, *spa*, *sdrC*, *sdrD*, *sdrE*, *clfA*, and *clfB*)^14^. It has been reported that this technique is quicker, cheaper and easier to use than PFGE and *spa*-typing, even though it is difficult to compare the results between laboratories. For this reason, it was recommended to use MLVF combined with a complementary typing method that provides portable data, like *spa*-typing or multi-locus sequence typing (MLST)^15^.

Very limited information is available about the *S. aureus* lineages causing diseases in Mexican cattle, as well as their antimicrobial resistances. One study classified the collected isolates using a phenotypic method based on the production of staphylokinase, the type of hemolysis displayed and the clotting of bovine plasma^16^. Another phenotypic typing study involved the classification of isolates by their hemolysis patterns^17^. In a third study, a genotypic characterization of different virulence factors was performed, including cell surface-associated and secreted proteins, and different classes of the accessory gene regulator *agr* were distinguished^18^. More recently, Valdez-Alarcón et al. performed MLST on *S. aureus* strains isolated from 3 different regions of Mexico^19^.

Most *S. aureus* isolates obtained from bovine mastitis have been shown to contain plasmid DNA^20^. The presence of differently sized plasmids was associated with the carriage of multiple antimicrobial resistances^21^. Staphylococcal plasmids have been shown to range from 1 to 60 kb in size. In *S. aureus*, two main classes of plasmids were identified, which contribute to the resistance against antimicrobials and/or virulence. All of these plasmids bear the features of conjugative or mobilizable plasmids^21^.

The aim of the present study was to investigate the diversity of 33 *S. aureus* isolates causing mastitis in cows from the Mexican Comarca Lagunera region by MLVF and *spa*-typing, as well as to determine the resistance of these strains against a variety of antimicrobials with possible links to the presence of plasmid DNA.

## Results

### Isolation of *S. aureus* mastitis strains

**Figure 1:**
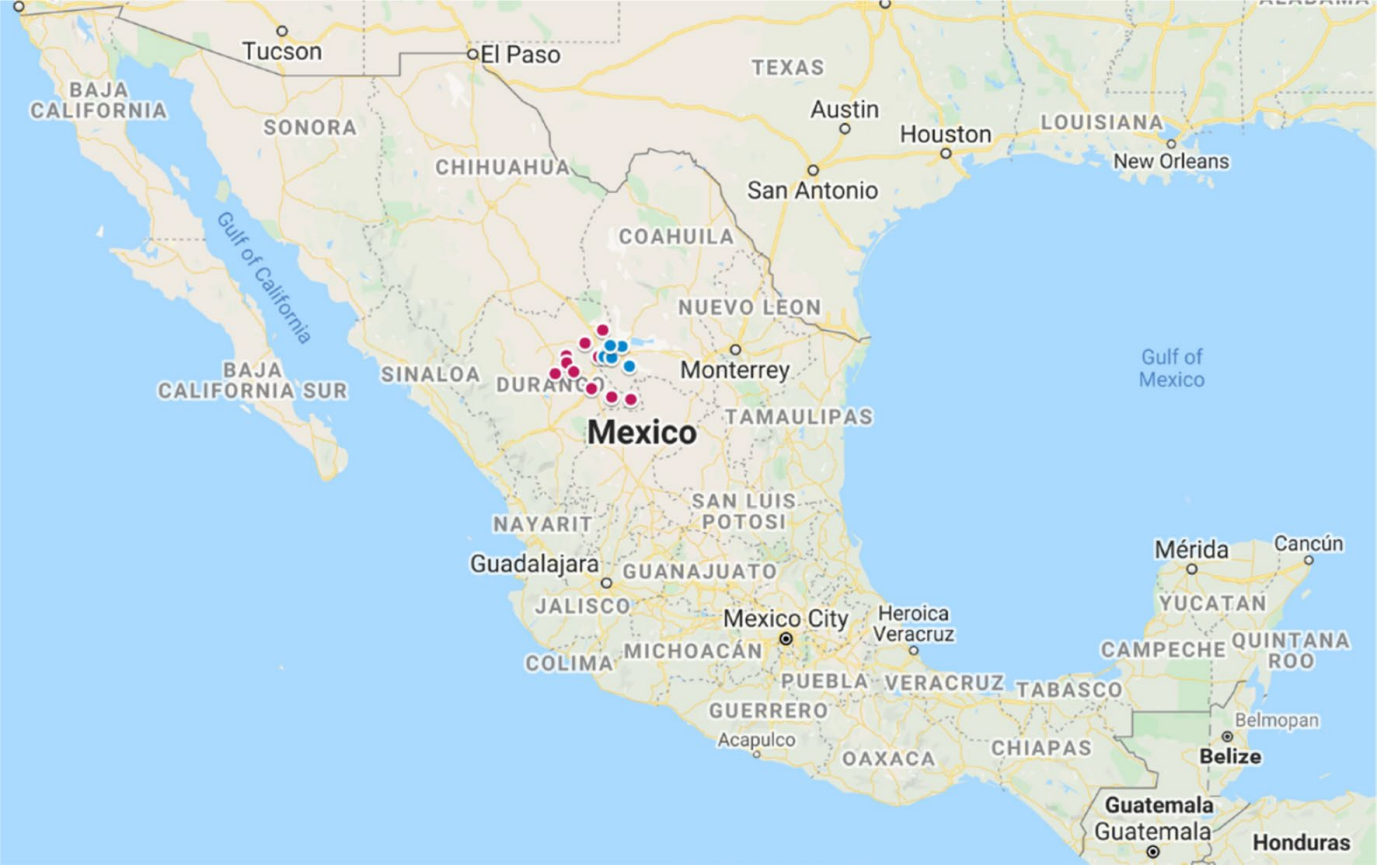
Map of Mexico indicating the Comarca Lagunera region from which the mastitis isolates were collected. The Comarca Lagunera region is marked with blue and pink dots. This region includes 5 municipalities of the Coahuila state (blue dots) and 11 municipalities of the Durango state (pink dots). The map was created from Google Maps (Map data Copyright 2020 Google) and edited in Microsoft PowerPoint 2016.

In total, 331 samples collected from milk of Holstein dairy cows (n = 226) and nasal swabs (n = 105) collected from their calves were collected from 14 farms in the region called Comarca Lagunera in Mexico (Fig. 1). The milk samples were taken from single quarters with clinical mastitis, where the criteria for mastitis were a somatic cell count > 400,000 SCC/mL. After a combination of Gram-staining, coagulase testing and MALDI-TOF analysis, 33 isolates of *S. aureus* obtained from individual cows at seven different farms (designated A-G) were identified (Table 1). To determine whether the potential presence of *S. aureus* strains resulted in transmission to calves, nasal samples (n = 105) were collected from calves that had contact with the cows with mastitis. All of the nasal swabs from calves tested negative for *S. aureus*, which suggests that transmission events between cows and calves were infrequent or remained undetected.

**Table 1:** Bacterial strains used in this study.

Isolates^a^	MLVF cluster; *spa*-type	Relevant phenotype or genotype	References
Newman		Clinical isolate	^48^
USA300		Community-acquired MRSA isolate	^49^
RF122		Mastitis-associated clinical isolate	^37^
B-1	A; t224	Mexican mastitis-associated isolate; orange-pinkish colonies	This study
C-1	B; t224	Mexican mastitis-associated isolates; white-yellowish colonies	
C-2			
C-3			
C-4			
C-5			
C-6			
C-7			
C-8			
C-9			
C-10			
C-11			
C-12		Mexican mastitis-associated isolates; orange-pinkish colonies	
C-13		Mexican mastitis-associated isolates; white-yellowish colonies	
C-14			
C-15			
E-1			
E-2			
E-3			
E-4			
E-5			
E-6			
E-7			
E-8			
E-9			
E-10			
E-11			
A-1	C; t416	Mexican mastitis-associated isolates; orange-pinkish colonies	
D-1			
F-1	C; t3196	Mexican mastitis-associated isolates; white-yellowish colonies	
F-2			
F-3			
G-1	D; t114		

^a^The letters A to G in the names of the isolates refer to the different farms from which they were collected.

### MLVF and PCR analysis

**Figure 2:**
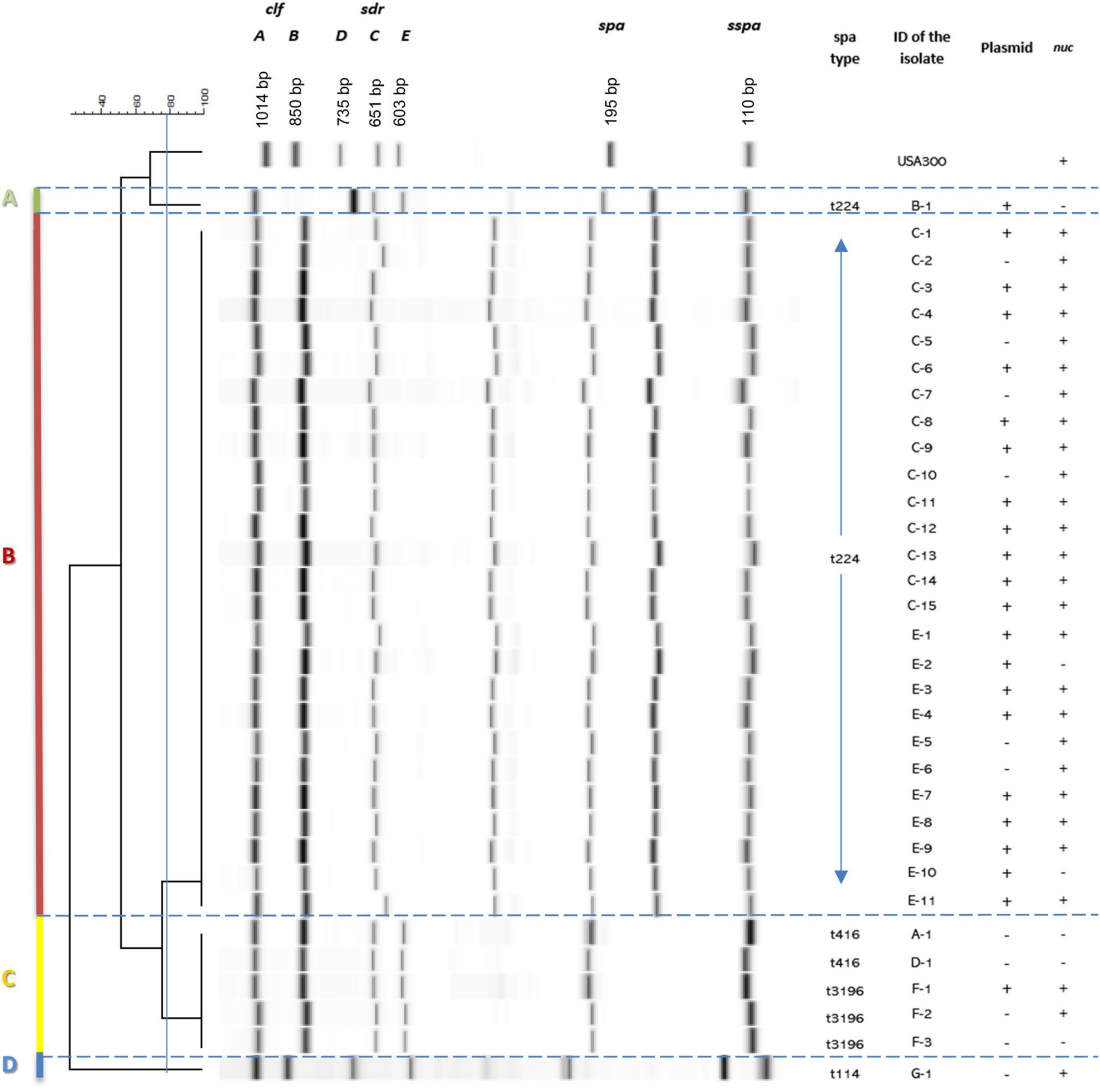
MLVF dendrogram of 33 *S. aureus* isolates from cows with mastitis. PCR fragments of seven VNTRs of *S. aureus* were separated on a Bioanalyzer 2100 with microfluidic DNA 7500 chips. The dendrogram was constructed in silico from CSV files of the Bioanalyzer runs using GelCompar II software (Applied Maths; https://www.applied-maths.com/gelcompar-ii). The PCR fragments (*clfA* [1014-bp], *clfB* [850-bp], *sdrD* [735-bp] *sdrC* [651-bp] *sdrE* [603-bp], *spa* [195-bp] *sspA* [110-bp]) obtained for the control DNA of *S. aureus* USA300 are indicated. For the generation of the dendrogram, a cut-off value of 81% was used, yielding a concordance of 0.980. The respective MLVF clusters (A-D), the respective *spa*-types, and the presence of the *nuc* gene and plasmid pSAM1 are specified. The Figure was created with Microsoft PowerPoint 2016.

The 33 mastitis-related *S. aureus* isolates were subjected to PCR analysis for the presence of genes of the micrococcal nuclease MN (*nuc*) and the methicillin resistance gene *mecA*. The *nuc* gene was detected in 27 isolates (Fig. 2). None of the isolates contained the *mecA* resistance gene. This matched with the antimicrobial susceptibility testing, because none of the isolates were resistant against cefoxitin or oxacillin. It was therefore concluded that all isolates were methicillin-susceptible *S. aureus* (MSSA)^22^.

For typing of the isolates, the total DNA was subjected to MLVF. The data obtained with the Bioanalyzer was used to create the MLVF dendrogram shown in Fig. 2. Altogether, the MLVF analysis of *S. aureus* isolates from milk samples revealed 4 different clusters, which were designated A (n = 1), B (n = 26), C (n = 5) and D (n = 1).

Judged by the expected lengths of the PCR fragments for the genes *sspA* (110-bp), *spa* (195-bp), *sdrE* (603-bp), *sdrC* (651-bp), *sdrD* (735-bp), *clfB* (850-bp), *clfA* (1014-bp) from the control strain *S. aureus* USA300, a clear variation in the lengths of several PCR fragments was observed among the four identified mastitis isolate clusters. While the PCR fragments representing the *sspA* and the *clfAB* genes appeared nearly identical for all isolates, the PCR fragments representing the *sdrDE* and *spa* genes showed clear variations between the clusters. Further, for all mastitis isolates the *spa* product was smaller compared to that of the control strain USA300. The amplified *spa* fragments from isolates belonging to clusters A and B appeared to be similar in length, while those for isolates in Cluster C seemed to be even smaller. Based on the band intensity, we assume that the *spa* and *sspA* PCR products had an identical size for isolates in cluster C. Lastly, the PCR products tentatively attributed to *sdrD* and *sdrE* seemed to be smallest for isolates from cluster B.

### *Spa*-typing

**Figure 3:**
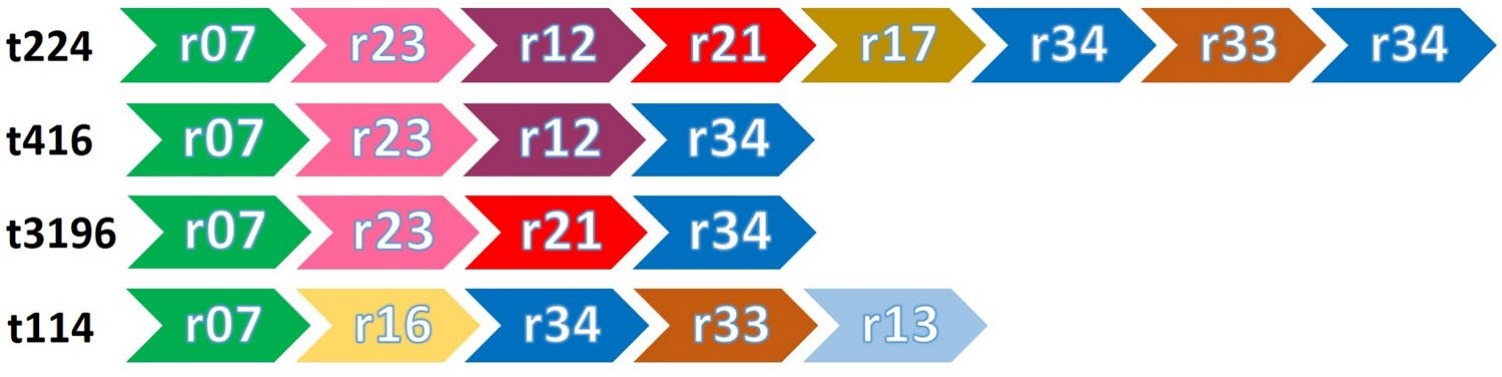
Schematic representation of the polymorphic VNTRs associated with identified *spa*-types. The order of identified VNTRs of the identified *spa*-types t224, t416, t3196 and t114 as detected in the present study isolates is schematically represented. The Figure was created with Microsoft PowerPoint 2016.

The 33 *S. aureus* isolates were also characterized by *spa*-typing, yielding 4 different *spa*-types (Figs. 2 and 3). *Spa*-type t224 was found to be the most common as it was assigned to 23 isolates. *Spa*-type t3196 was assigned to 3 isolates, t416 to 2 isolates and t114 was identified for only one isolate. Notably, all isolates belonging to the MLVF clusters A and B had the *spa*-type t224, whereas *spa*-type 114 was unique for MLVF cluster D. In contrast, isolates belonging to MLVF cluster C had either *spa*-type t3196 or t416. The variation in the number of repeats within the *spa* genes of the different isolates was in agreement with the variations in length that were observed for the respective *spa-*specific PCR fragments in the MLVF (Figs. 2 and 3). When comparing the composition of the different types of identified repeats within the *spa* gene, it seems that isolate G-1 (t114) includes two repeat sequences (r16 and r13) that are not present in the *spa* genes of the other mastitis isolates (Fig. 3).

### Western blotting analysis

**Figure 4:**
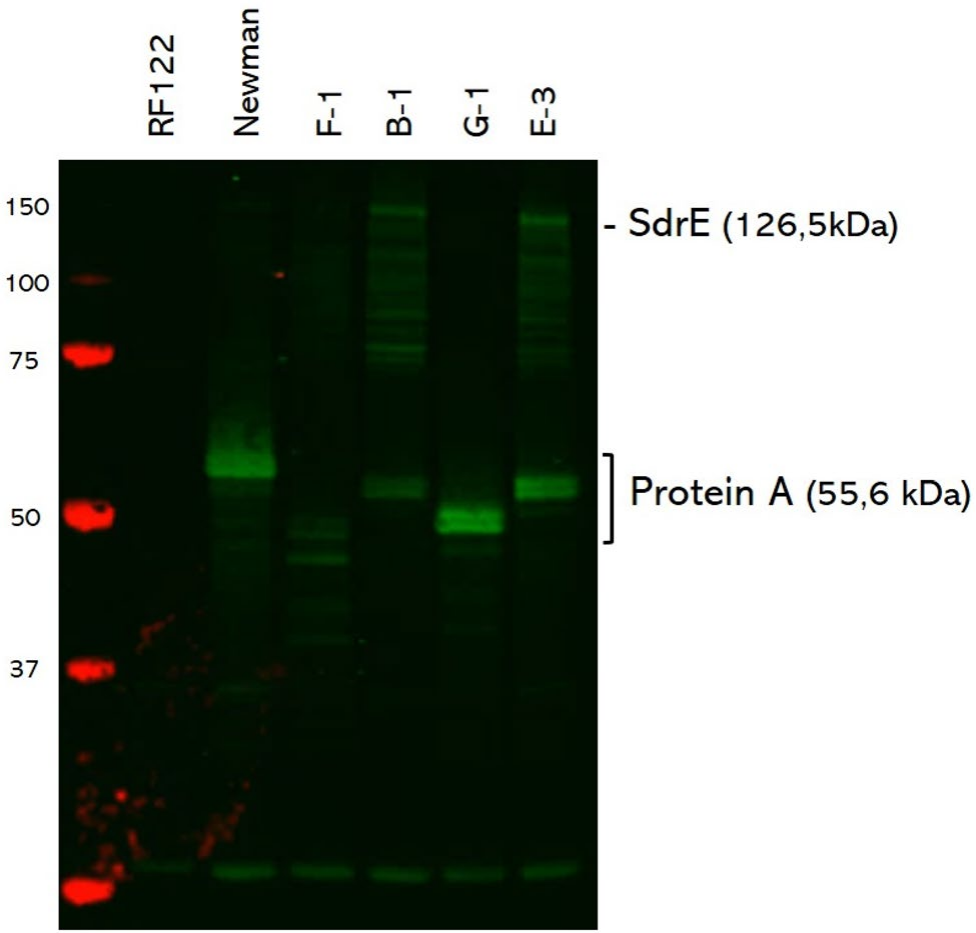
Western blotting analysis to detect the presence of SdrE and protein A. Proteins in the cellular fractions of *S. aureus* strains RF122 and Newman, and the mastitis isolates F-1, B-1, G-1 and E-3 were separated by LDS-PAGE and transferred to a nitrocellulose membrane. To visualize the presence of SdrE and protein A, the membrane was incubated with SdrE-specific rabbit antibodies (1:5000) and, subsequently, with an IRDye800CW-labelled goat anti-rabbit IgG secondary antibody (1:10,000). Note that protein A is detected by its capacity to bind rabbit IgG. The sizes of the marker proteins are indicated in kDa at the left side of the membrane. On the right side, the position of SdrE- and protein A-specific protein bands is marked. The complete original Western blot is presented as Supplemental Fig. S2. The Figure was created with Microsoft PowerPoint 2016.

To verify the observed size differences of the *spa* and *sdrE* PCR products in the MLVF pattern, a Western blotting analysis was performed to detect the respective protein A and SdrE proteins. For this analysis the covalently cell wall-bound proteins of 6 different mastitis isolates were extracted. As controls, the *S. aureus* strain Newman, which was originally isolated in 1952 from a human infection, and the mastitis-related *S. aureus* strain RF122 were used. The strain Newman is known to produce protein A (55.6 kDa) and SdrE (126.5 kDa), while the RF122 strain lacks expression of protein A. From the Mexican mastitis isolates, one strain of each MLVF cluster was selected. Specifically, these included isolates F-1 (t3196), B-1 (t224), G-1 (t114) and E-3 (t224). Using a polyclonal antibody against SdrE, both the expression of SdrE and protein A could be determined by Western blotting (Fig. 4). In the case of protein A, this relates to the fact that it efficiently binds the Fc region of rabbit polyclonal antibodies. The results of the blot are congruous with the VNTR repeats of the different *spa*-types. The VNTR repeats of the isolates B-1 and E-3 with *spa-*type t224 (MLVF clusters A and B, respectively) were equal in size and slightly smaller than those of the control strain Newman. For isolate G-1 with *spa*-type 114 (MLVF cluster D), a smaller size for protein A was detected. The smallest form of protein A was detected for isolate F-1 with spa type t3196 and belonging to the MLVF cluster C (Figs. 3 and 4).

Unexpectedly, no protein band corresponding to SdrE was detected for strain RF122 (Fig. 4). In the sample of strain Newman a clear SdrE signal at the expected height of 126.5 kDa was detected together with multiple degradation bands. Similar patterns were observed for isolates F-1, B-1 and E-3, indicating that these isolates do express SdrE. Yet, no SdrE signal was detectable for isolate G-1 (Fig. 4), indicating that it does not express the SdrE protein, even though a possible *sdrE* PCR fragment appeared to be detectable in the MLVF analysis (Fig. 2).

### Antimicrobial resistance profiling

To determine possible variations in antimicrobial resistance and possible association of resistance phenotypes with the MLVF and/or *spa*-types of the investigated mastitis isolates, all 33 isolates were examined for antimicrobial susceptibility. The analysis of antimicrobial susceptibility revealed that for the antimicrobials with clinical breakpoints all isolates were susceptible to erythromycin, oxacillin, and tetracycline. Thirty-two isolates were resistant to benzylpenicillin (97%). Only strain G-1 (3%) was susceptible to all tested antimicrobials. Lastly, all isolates tested negative in the screen for cefoxitin resistance.

### Plasmid profiling and sequence analysis

**Figure 5:**
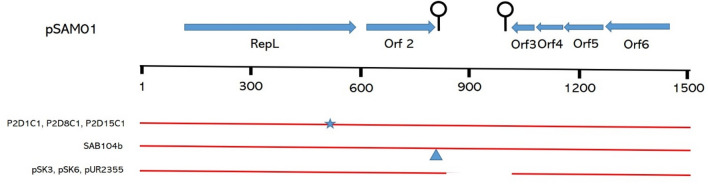
Schematic representation of open reading frames on plasmid pSAM1. The relative positions of open reading frames (*repL*, 2, 3, 4, 5 and 6) are marked by arrows. Predicted stem-loop structures are indicated by lollypops. Regions of sequence identity between different plasmids are indicated by red lines, in which mutations (*) and insertions (∆) are marked. The names of the homologous plasmids are indicated on the left. The Figure was created with Microsoft PowerPoint 2016.

To determine the presence of plasmids, plasmid DNA was isolated from all mastitis isolates. Using agarose gel electrophoresis it was shown that 22 out of the 33 isolates contain a plasmid of identical size (Supplemental Fig. S1). No plasmid DNA was detected in strains with the *spa*-types t114 and t416. On basis of the electrophoresis results, the plasmid DNA seemed to be of the same size in all strains. The plasmid DNA extracted from the isolates F-1 (t3196), E-3 (t224) and C-11 (t224) was sequenced. Interestingly, the respective plasmid sequences were found to be identical, consisting of 1508-bp. Accordingly, the respective plasmid was named pSAM1. Searches for open reading frames (ORF) combined with Blast analyses showed the presence of several ORFs in pSAM1 (Fig. 5).

The plasmid sequences showed a G + C content of 32.6 mol%, which is in agreement with the G + C content determined for *S. aureus* (33 mol%)^23^. Based on DNA sequence similarity, plasmid pSAM1 can be classified as a rolling circle replicating (RCR) plasmid that belongs to the pSN2 Family^21^. In total, six ORFs were identified on pSAM1 (Fig. 5). The largest identified *orf* of pSAM1 encodes a protein of 154 amino acid (AA) residues which, on the basis of sequence similarity, corresponds with the replication protein RepL. Downstream of the *repL* gene, the *orf2* gene is located, which encodes a protein of 62 residues with unknown function. An inverted repeat with a ∆G° of − 12.6 kcal/mol that may function as a rho-independent terminator is located downstream of *orf2*. 136-bp downstream of this stem-loop another stem-loop with a ∆G° of − 10.2 kcal/mol is located, which could function as a terminator for the *orf3.* Blast analysis shows that the sequence similarity with other plasmids stops directly after either of these two stem loops, suggesting that they are potential sites for recombination. Blast analyses also showed that in *S. capitis* a nucleotide sequence is present encoding a protein of 73 residues with unknown function, which overlaps with *orfs 3, 4* and *5*^24^. Also, in other *S. aureus* plasmids like pSK3, pSK6 and pUR2355, mutations have been identified for this ORF (Fig. 5), suggesting that the presently identified *orfs 3, 4* and *5* do not encode a functional protein in *S. aureus*. The last *orf*, *orf6*, has a G + C content of 46%, which is much higher than the average G + C content of 32.6% observed for the rest of the plasmid.

Overall, plasmid pSAM1 shows a high degree of sequence similarity with the plasmids P2D1C1, P2D8C1 and P2D15C1 that were previously identified in other *S. aureus* strains^25^. Compared to these plasmids small mutations are located in the *repL* gene. The overall homology with a plasmid from *S. aureus* strain SAP104B (1552-bp) was also very high although the SAP104B-derived plasmid contained some extra nucleotide sequences in between *orf3* and the downstream located stem loop. On basis of its *repL* gene plasmid pSAM1 was grouped in *rep* family 10b^26^.

## Discussion

In the present study, we describe the diversity of 33 *S. aureus* isolates causing mastitis in Mexican cows by MLVF and *spa*-typing, as well as their susceptibility for a range of commonly used antimicrobials.

Our typing analysis shows that the *S. aureus* isolates associated with mastitis group into four MLVF clusters, which overlap with the determined *spa*-types. The predominantly encountered *spa*-type t224 was determined for all isolates of the MLVF clusters A and B. In recent studies, this *spa*-type has been reported for *S*. *aureus* isolates from milk of cows with intra-mammary infections in European countries, China, Japan and Egypt^9,27,28^. The present identification of mastitis-associated *S. aureus* isolates with *spa*-type t224 in Mexico implies that this staphylococcal type has now been detected on all continents except Australia. However, in other studies, t224 was not the predominantly encountered *spa*-type. Of note, the *spa-*type t224 is frequently observed for isolates belonging to the ST97 group that is known to be carried by cows, humans and swine. In swine, such isolates are occasionally MRSA^29^. Whether the isolates used in this study also belong to the ST97 remains to be investigated. The MLVF cluster C includes the *spa*-types t416 and t3196. *S. aureus* isolates with *spa*-type t416 have been reported in Austria and China, mainly for human MRSA isolates^30,31^. According to the Ridom Spa Server (https://spaserver.ridom.de/), *S. aureus* isolates with the *spa*-type t3196 have been identified in Belgium, Denmark, and the Netherlands. However, no information is currently available concerning the respective host species, or whether these t3196 isolates were associated with bovine mastitis. The MLVF cluster D is formed by a singleton with *spa*-type t114, which has been described for *S. aureus* isolates from the milk of cows with mastitis in Brazil and China^32,33^.

The *nuc* gene, which encodes a thermostable nuclease, has been described as a specific marker for direct detection of *S. aureus* involved in infection of humans^34^. Nonetheless, in 4 of the 33 presently investigated *S. aureus* isolates the *nuc* gene was apparently not present. Interestingly, Javid et al*.* reported that *nuc* could only be detected in a fraction of *S. aureus* isolates collected from cows with mastitis in India^35^. These observations are intriguing, as it has been proposed that the micrococcal nuclease is involved in the evasion of the human immune system, by preventing the capture and elimination of *S. aureus* in neutrophil extracellular traps^36^.

It was previously reported that the *spa*, *clfA* and *sdrC* genes of the bovine mastitis-related *S. aureus* strain RF122 are pseudogenes, because of the presence of premature stop codons^37^. This phenomenon was not observed for the *spa* genes of the presently investigated *S. aureus* isolates from Mexico, because protein A was detectable for all isolates by Western blotting. On the other hand, RF122 contains a complete s*drE* gene, which was confirmed in a Western blot using an antibody against the region A of the SdrE protein of *S. aureus* strain Newman^38^. The sequence similarity of region A in the SdrE proteins of strains RF122 and Newman is only 76%. This difference in the sequence could be the reason that SdrE was not detected by Western blotting in the sample for covalently cell wall-bound proteins of *S. aureus* RF122. Another possible explanation could be the use of whey permeate as a growth medium for *S. aureus* in the present study, which may have lowered the expression of SdrE in the RF122 strain.

Antimicrobial resistance testing showed that the majority of the isolates (32/33) were resistant to penicillin. This is not surprising, as this antimicrobial is the first option to treat bovine mastitis. In many dairy farms it is common practice to use intramammary infusions of antimicrobials as a prophylactic approach to prevent and control mastitis in all dairy cows during the dry period, primarily with penicillins, cephalosporins, or other beta-lactam drugs^6^.

In 67% of the investigated isolates, the presence of a single plasmid was detected. Based on agarose gel electrophoresis and sequencing data, it seems that all plasmid-bearing strains contained the same plasmid, which was named pSAM1. Sequence analysis of the *repL* gene showed that pSAM1 belongs to the pSN2 family, which encompasses the smallest RCR plasmids identified in staphylococci^21^. The presence of pSAM1 was evidently not linked to any of the MLVF clusters, a *spa*-type or a specific antimicrobial resistance. Besides the putative replication protein RepL, no function has so far been assigned to any of the five other proteins encoded by pSAM1. The high prevalence of pSAM1 amongst the presently investigated *S. aureus* isolates could suggest an association of this plasmid and its encoded proteins with bovine colonization or even mastitis. However, the latter remains to be investigated, preferably in the context of follow-up studies on possible adaptations that allowed the present mastitis isolates to thrive in the bovine mammary gland.

## Materials and methods

### Sample collection and selection

Cases of mastitis were identified using the California Mastitis Test (CMT), which is a simple on-farm method to indirectly indicate the level of the somatic cell count (SCC)^39^. Two hundred and twenty six individual quarter milk samples were collected in 2015–2016 from 14 different farms in the Comarca Lagunera region in Mexico. Foremilk was discarded after which a little amount of milk was drawn in the CMT paddle. An equal amount of the CMT reagent was added and gently mixed. After a few seconds, the reaction was scored following the scale described by Easterday et al.^39^. The weakly positive samples with 400,000 to 1,000,000 SCC/mL (mixture is slightly mucous, but still without gel formation), positive samples with 800,000 to 5,000,000 SCC/mL (distinct gel formation), and the samples with more than 5,000,000 SCC/mL (strongly positive with strong gel formation that adheres to the paddle) were collected for plating. For transmission analysis single nasal swabs were collected from 105 calves of these cows.

### Identification of isolates and culture conditions

All milk samples and nasal swabs collected from calves were directly plated at the farm on mannitol salt agar (Becton Dickinson de Mexico). Subsequently, the plates were incubated at 37 °C overnight to determine the presence of bacteria. From each plate one potential *S. aureus* colony was selected, depending on the following colors: golden-yellow, creamy-yellow, yellowish, orange, pinkish and white (Table 1). After Gram-staining, all the Gram-positive cocci were subjected to the catalase^40^ and Voges Poskauer^41^ tests. Finally, all colonies that tested potentially positive as *S. aureus* in the biochemical tests were collected and shipped in mannitol salt agar to the University Medical Center Groningen, the Netherlands.

The potential *S. aureus* isolates were cultured on Blood Agar (BA) plates containing 5% sheep blood and aztreonam (Media products, Groningen, the Netherlands). Firstly, a quick screen of the strain was done using the Pastorex Staph Plus test (Bio-Rad, Marnes-la-Coquette, France). To select and identify *S. aureus* strains*,* single colonies were analyzed by matrix-assisted laser desorption ionization-time of flight mass spectrometry (MALDI-TOF MS) with a Microflex LT Biotyper (Bruker Daltonics, Bremen, Germany) as previously described^42^. Only *S. aureus* strains with log scores ≥ 2 were selected. Thirty-three isolates were positively identified as *S. aureus.* The isolates were cultured overnight in tryptic soy broth (TSB, Oxoid, Hampshire, UK) at 37 °C with shaking (250 rpm) and, subsequently, stored in 12% glycerol (Sigma-Aldrich, Zwijndrecht, the Netherlands) at −80 °C for further analyses.

### Antimicrobial susceptibility testing

Antimicrobial susceptibility was measured with the VITEK 2 system (ID. Card: AST-GP69, bioMerieux Corporate, Marcy l’Etoile, France) in accordance with the manufacturer’s instructions. For quality control of the card, the strain *S. aureus* ATCC 29,213 was used as suggested by the supplier. The following antimicrobials were evaluated: benzylpenicillin, cefoxitin, enrofloxacin, erythromycin, kanamycin, oxacillin and tetracycline. The minimum inhibitory concentrations (MIC) obtained from the VITEK analysis were validated using the Advanced Expert System following the Clinical and Laboratory Standards Institute (CLSI) of the veterinary guidelines^43^, which employed the human interpretive data taken from the CLSI M100-S series^44^. The MIC breakpoints are specific for *S. aureus* udder isolates and are expressed as μg/ml in parentheses. These breakpoints are for benzylpenicillin (Susceptible [S] ≤ 0.12, Resistance [R] ≥ 0.25); erythromycin (S ≤ 0.5, R ≥ 8); oxacillin (S ≤ 2, R ≥ 4) and tetracycline (S ≤ 4, R ≥ 16). For cefoxitin only negative or positive test results were obtained. For the veterinary antimicrobials enrofloxacin and kanamycin, interpretative data are not available for mastitis-associated *S. aureus*. All results are presented in the Supplementary Table S1.

### MLVF and PCR analysis

Total DNA was extracted from *S. aureus* colonies picked from BA plates by bead-beating according to Glasner et al*.*^14^.

MLVF typing was performed following the adjusted protocol described by Sabat et al.^15^ and Glasner et al*.*^14^*.* Briefly, 1 μL of total DNA was subjected to a multiplex PCR of seven VNTRs of *S. aureus* (*sdrC, sdrD, sdrE, clfA, clfB, sspA* and *spa*) (Eurogentec, Maastricht, the Netherlands). Subsequently, 1 µL aliquots of the resulting amplicons were separated employing a Bioanalyzer 2100 (Agilent Technologies, Palo Alto, USA) with microfluidic DNA 7500 chips following the manufacturer’s instructions. The strain USA 300 was used as the technical control in each Bioanalyzer run to ensure the reproducibility of the data. Data was analyzed with the GelCompar II software (Applied Maths, Kortrijk, Belgium). For this purpose, the data generated in the Bioanalyzer were imported as CSV files. The position tolerance and optimization were set at 0.9% and 0.5% respectively, as with these settings the control isolate (USA300) displayed identical MLVF banding patterns. To calculate the pairwise similarity coefficients, the dice formula was used. A dendrogram was created with the unweighted pair group method using average linkages (UPGMA). A cut-off of 81% was set to allow a differentiation between clusters. Identical banding patterns were assigned to the same subtype; when one or more bands differed in the MLVF pattern, they were assigned to different clusters.

All *S. aureus* strains were also screened for the presence of the *mecA* gene encoding methicillin resistance and the *nuc* gene for the micrococcal nuclease MN produced by *S. aureus*, as previously described^34,45^.

### *Spa*-typing

*Spa*-typing was performed as described by Harmsen et al*.*^13^ using the Ridom Staph Type software version 2.2.1 (Ridom GmbH, Würzburg, Germany). An ABI Prism 3130 genetic analyzer (Applied Biosystems, Foster City, USA) was used to obtain the DNA sequences.

### Plasmid sequencing

Plasmid DNA was extracted using the Innu PREP Plasmid Mini Kit (Analytik Jena, Jena, Germany) following the special protocol of the supplier, where in the second step the cell pellet was resuspended in resuspension buffer with added lysostaphin (final concentration 200 µg/mL; AMBI Products, Lawrence, USA) and lysozyme (final concentration 3 mg/mL; Sigma‐Aldrich). The bacteria were incubated at 37 °C for 10–30 min until complete lysis was obtained. Plasmid DNA was analyzed using 1.0% agarose gels. Nucleotide sequence analyses of the plasmids were performed by an Illumina MiSeq system generating paired-end reads of 300 bp (Illumina, CA, USA). De novo assembly of paired-end reads was performed using the CLC Genomics Workbench v11.0.1 (QIAGEN, Hilden, Germany). Homology comparisons were performed using the basic logical alignment tool (https://blast.ncbi.nlm.nih.gov/Blast.cgi). Dyad symmetries, Delta G calculation and Open Reading Frames (ORFs) were determined using the program CloneManager 9 (Sci-Ed software, Westminster, Colorado, USA). The GenBank accession number of plasmid pSAM1 is 2,388,794.

### Western blotting

*S. aureus* strains were grown in TSB overnight at 37 °C, 250 rpm. Bacterial cultures were diluted to an optical density at 600 nm (OD_600_) of 0.05 in 4% whey permeate medium containing 0.2% casein hydrolysate (ICN Biochemicals, Ohio, USA) and 1.9% glycerolphosphate (SIGMA Life Science, Missouri, USA). Growth was continued under the same conditions until the exponential phase (OD_600_ = 0.5) was reached. After this pre-culture, the cells were diluted once more in whey permeate medium^46^ and growth was continued for 2 h. Bacterial culture samples of 2 mL were pelleted and washed once in phosphate-buffered saline (PBS, Media products). The obtained cell pellet was disrupted with 0.1 µM glass beads in PBS with a protease inhibitor cocktail (complete, Mini, EDTA-free, Roche) in a Precellys 24 homogenizer (Bertin Technologies, Saint Quentin en Yvelines Cedex, France). After centrifugation, cell wall fragments were resuspended in 4% sodium dodecyl sulphate (Sigma-Aldrich), incubated for 3 min at 100 °C, and washed six times with PBS^47^. Afterwards, the cell wall fragments were incubated in 50 mM Tris (pH 7.5, Sigma-Aldrich) with lysostaphin (final concentration 200 µg/mL) and protease inhibitor cocktail (Roche) for 3 h at 37 °C. The original Western blotting image is presented as Supplemental Fig. S2.

Covalently cell wall-bound proteins were separated by lithium dodecyl sulphate (LDS)-PAGE using NuPAGE gels (Life Technologies, California, USA) and subsequently transferred to a nitrocellulose membrane (Protran nitrocellulose transfer paper, Whatman, Germany). Membranes were incubated with polyclonal rabbit antibodies against SdrE (1:5,000) and thereafter with an IRDye800CW-labelled goat anti-rabbit IgG secondary antibody (1:10,000; LI-COR Biosciences, Lincoln, USA). Finally, the membranes were scanned with an Odyssey Infrared Imaging System (LI-COR Biosciences) for fluorescence at 800 and 700 nm.

### Biological and chemical safety

*S. aureus* is a biosafety level 2 (BSL-2) microbiological agent and was accordingly handled following appropriate safety procedures. All experiments involving live *S. aureus* bacteria were performed under appropriate BSL-2 containment as approved by the biological safety officers of the University Medical Center Groningen (UMCG). All chemicals and reagents used in this study were handled according to the local and international guidelines and protocols for safe usage and protection of the environment.

### Ethics

For the isolation of bacteria from waste milk samples of mastitic cows and for the non-invasive nasal swabs from calves of mastitic cows, in Mexico no ethical permission was required at the time of sampling. Since all investigated bacteria were collected in Mexico, no ethical approval was required for the investigations performed at the University Medical Center Groningen (UMCG). The research project was performed with adherence to the Nagoya Protocol on Access and Benefit Sharing (ABS).

## Supplementary Information

Supplementary Information 1.

Supplementary Information 2.
