# Supplementary material for: Molecular typing and antimicrobial resistance profiling of 33 mastitis-related Staphylococcus aureus isolates from cows in the Comarca Lagunera region of Mexico
Source: Sci Rep. 2021 Mar 25;11:6912. doi: 10.1038/s41598-021-86453-2 (PMC7994548; doi:10.1038/s41598-021-86453-2)
Supplement: Supplementary file 1 — Supplementary Information 1. [file 41598_2021_86453_MOESM1_ESM.pdf]

Supplementary Figures 1 and 2

Molecular typing and antimicrobial resistance profiling of 33 mastitis-related *Staphylococcus aureus* isolates from cows in the Comarca Lagunera region of Mexico

Y. Mora-Hernández[1], E. Vera Murguía[1], J. Stinenbosch[1], P. Hernández Jauregui[2], J. M. van Dijl[1, #] and G. Buist[1]

[1] University of Groningen, University Medical Center Groningen, Department of Medical Microbiology, Hanzeplein 1, P.O. Box 30001, 9700 RB Groningen, the Netherlands

[2] Cyta Labs, Puebla, Mexico

[#]**Corresponding author**: Jan Maarten van Dijl, Department of Medical Microbiology, University of Groningen, University Medical Center Groningen, Hanzeplein 1, P.O. Box 30001, 9700 RB Groningen, the Netherlands. Phone: +31-50-3615187; Fax: +31 50 361 9150. E-mail: j.mvan.dijl01@umcg.nl

Running title: Typing Mexican *S. aureus* mastitis isolates

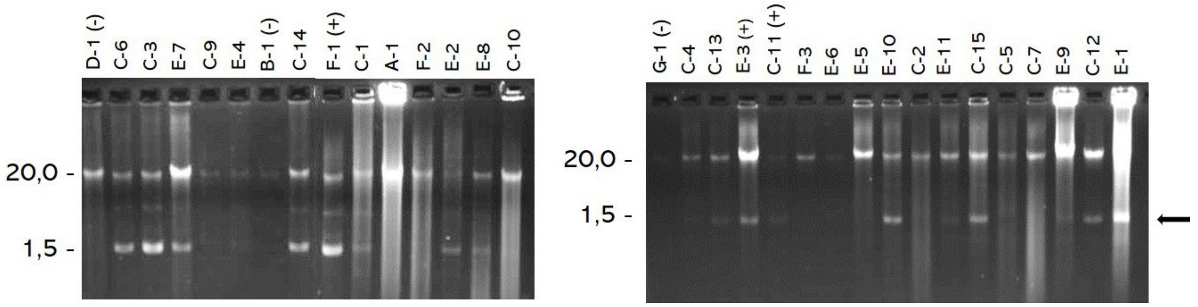

Supplementary Figure 1: Presence of pSAM1 in *S. aureus* isolates from cows with mastitis. The presence of plasmids in the different *S. aureus* isolates was assessed by agarose gel electrophoresis. The presence of a DNA band that is diagnostic for pSAM1 is marked by an arrow. Names of the investigated strains are indicated on top of the gel image. On the left of each gel, the sizes of 2 marker DNA fragments are indicated in kb. The Figure was created with Microsoft PowerPoint 2016.

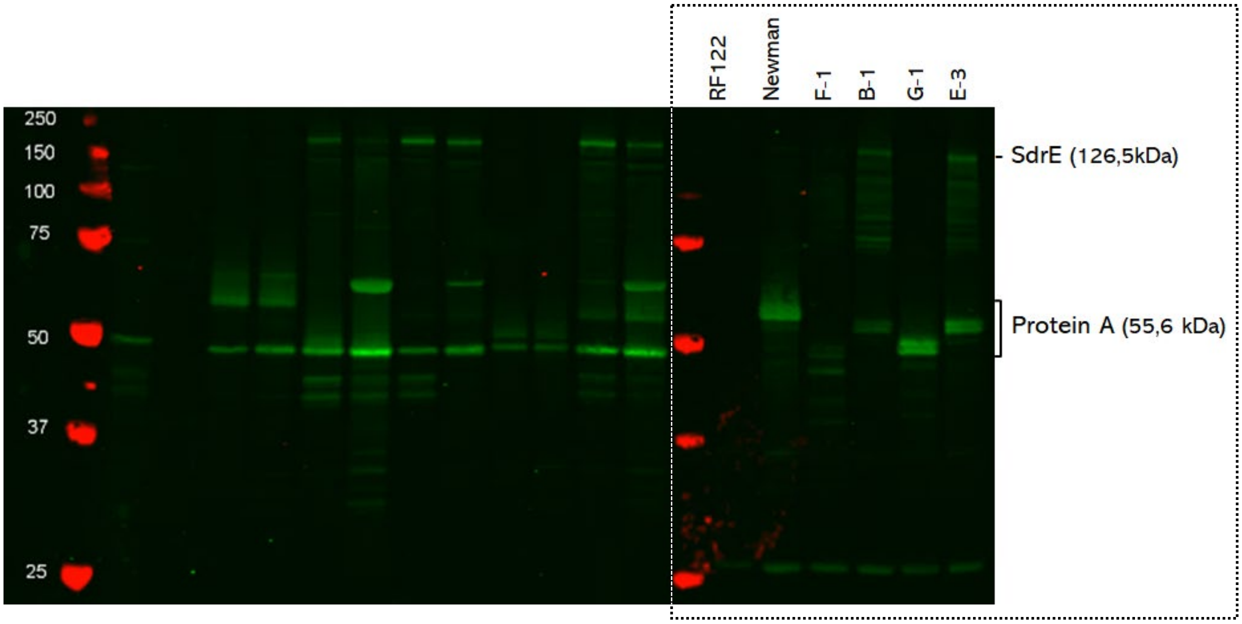

Supplementary Figure 2: Original Western blot for Figure 4. The dotted box marks the segment of the Western blot that was used to create Figure 4. The Figure was created with Microsoft PowerPoint 2016.
